# LC-ESI/QTOF-MS Profiling of Chicory and Lucerne Polyphenols and Their Antioxidant Activities

**DOI:** 10.3390/antiox10060932

**Published:** 2021-06-08

**Authors:** Yasir Iqbal, Eric N. Ponnampalam, Hafiz A. R. Suleria, Jeremy J. Cottrell, Frank R. Dunshea

**Affiliations:** 1 School of Agriculture and Food, Faculty of Veterinary and Agricultural Sciences, The University of Melbourne, Parkville, VIC 3010, Australia; yasir.iqbal@student.unimelb.edu.au (Y.I.); hafiz.suleria@unimelb.edu.au (H.A.R.S.); jcottrell@unimelb.edu.au (J.J.C.); 2 Animal Production Sciences, Agriculture Victoria Research, Department of Jobs, Precincts and Regions, Bundoora, VIC 3083, Australia; Eric.Ponnampalam@agriculture.vic.gov.au; 3 Faculty of Biological Sciences, The University of Leeds, Leeds LS2 9JT, UK

**Keywords:** chicory, lucerne, polyphenols, extraction, antioxidants, LC-ESI/QTOF-MS, HPLC-PDA

## Abstract

Chicory and lucerne are used as specialised forages in sheep or dairy production systems in some parts of the world. Recently, these plants are gaining attention as raw materials in the search for natural antioxidants for use in animal feeds, human foods and nutraceutical formulations. The antioxidant potential of these plants is credited to polyphenols, a subgroup of phytochemicals. Therefore, phenolic characterisation is an essential step before their use as ingredients in animal feeds, human food or nutraceutical preparations. In this study, we performed qualitative and quantitative analysis of polyphenols in chicory and lucerne. Profiling of polyphenols from chicory and lucerne was performed by LC-ESI/QTOF-MS with a total of 80 phenolic compounds identified in chicory and lucerne. The quantification of polyphenols was achieved by high performance liquid chromatography, coupled with a photo diode array (HPLC-PDA). Chicoric acid was the major phenolic acid found in chicory, with the highest concentration (1692.33 ± 0.04 µg/g DW) among all the polyphenols quantified in this study. 2-hydroxybenzoic acid was the major phenolic acid found in lucerne, with the highest concentration of 1440.64 ± 0.04 µg/g DW. Total phenolic, flavonoids and total tannin contents were measured, and the antioxidant potential was determined by 2,2-Diphenyl-1-picrylhydrazyl, Ferric Reducing Antioxidant Power, 2,2-Azino-bis-3-ethylbenzothiazoline-6-sulfonic Acid, Hydroxyl (OH^−^) Radical Scavenging Activity, Chelating Ability of Ferrous Ion (Fe^2+^) and Reducing Power (RPA) assays. Both chicory (8.04 ± 0.33 mg AAE/g DW) and lucerne (11.29 ± 0.25 mg AAE/g DW) showed high values for Hydroxyl (OH^−^) Radical Scavenging Activity. The current study allowed us to draw a profile of polyphenols from chicory and lucerne. They provided a molecular fingerprint useful for the application of these plant materials in human foods, animal feeds and pharmaceutical formulations.

## 1. Introduction

In recent years, attempts to find natural antioxidants for use in animal feeds, human foods and medicines have increased, in order to replace their synthetic counterparts due to their harmful effects to body and consumer choice. Therefore, natural plant materials gain popularity in various industrial sectors like human food, animal feed, cosmetics and pharmaceuticals. Selected plant materials can contribute to nutrition and natural therapeutics for the cure of various ailments, such as inflammation and oxidative stress due to the presence of bioactive compounds like polyphenols. Polyphenols are bioactives formed in plants as defensive compounds against ultraviolet radiation or aggression by pathogens [1]. These phenolic compounds possess various health-promoting properties, for example being antioxidative, anti-inflammatory, antiaging, antibacterial, antidiabetic and anti-mutagenic [2,3,4].

These health-promoting effects of polyphenols are ascribed to their strong antioxidant potential and ability to scavenge free radicals, thereby delaying or avoiding lipid and protein oxidation in plant and animal tissues [5,6,7]. Besides their importance in physiological systems, polyphenols also gain importance as alternatives to synthetic antioxidants and preservatives in food [8]. Therefore, identification and complete profiling of polyphenols from plants can provide valuable information for their optimum use in various industrial sectors, such as livestock production, food production and cosmetics, particularly in pharmaceutical formulations and functional foods [9].

Polyphenols can be extracted from plant materials by various methods using different solvents and extraction techniques. The extraction procedure is of prime importance in the analysis of polyphenols [10]. Hence, the use of an appropriate extraction technique is vital for obtaining accurate results in terms of qualitative and quantitative determination. Various methods are applied to optimise the extraction process. However, a solid-liquid extraction method using different solvents is considered more appropriate for polyphenols [11]. Various techniques can also be used to characterise polyphenols. Recently, high performance liquid chromatography (HPLC), coupled with mass spectrometry (MS), has evolved as a new technique [12] that allows more accurate identification and characterisation of single and complex phenolic compounds and other metabolites. This technique is highly sensitive for identifying small molecules with specific masses in a complex mixture, and identifies compounds based on mass-to-charge ratios [10].

Chicory (*Cichorium intybus*) and lucerne (*Medicago sativa*) have strong antioxidant potential. Chicory and lucerne forages are used in dairy production and sheep meat production systems in southern Australia and many parts around the world, due to their high protein, soluble carbohydrates and bioactive compounds. Both forages are perennials with three-to-four years of persistence in the field, with high dry matter production during spring and early summer seasons in Southern Australia, when the weather is warm with adequate water available in the soil. Chicory and lucerne can deliver high forage quality due to their deep tap root systems, resulting in a consistent growth rate with greater assimilation of nutrients (proteins, soluble carbohydrates, vitamins) in the vegetative parts, even with less water availability in the soil. They can also be stored as silage, haylage or hay (lucerne) when produced in excess amounts. These preserved feeds can be used as alternative feeds or supplements during dry seasons to increase the growth and productivity (milk and meat) of farm animals, when the availability of traditional pastures is low. Chicory possesses antioxidant, anti-parasitic, anticancer, antihepatotoxic, antibiotic and anti-inflammatory actions. Lucerne has been used in the treatment of digestive tract ailments and problems of the circulatory and immune systems in traditional (folk) medicine [13,14]. Detoxification and anticarcinogenic properties, particularly in the alimentary tract, have also been attributed to lucerne use [13]. These plants’ health-promoting activities are ascribed to their secondary metabolites like phenolic acids, flavonoids, coumarins and tannins [15,16]. The presence of phenolic acids and flavonoids has been reported in chicory and lucerne [17,18,19]. Based on their high phenolic composition, chicory and lucerne have a high therapeutic potential that has not been fully understood, as there have been few studies characterising the bioactive components of these plants. Therefore, this study was conducted to provide more reliable and accurate information about the phenolic contents of chicory and lucerne for utilisation in foods, feeds and medicinal formulations.

## 2. Materials and Methods

### 2.1. Plant Material

Chicory (*Cichorium intybus* var. Commander) and lucerne (*Medicago sativa* var. Sardi 7 series II) were acquired from Hamilton Research Station, Agriculture Victoria Research, Department of Jobs, Precincts and Regions, 915 Mt. Napier Road, Hamilton, VIC 3300, Australia. Vegetative (leaves and stems) parts of chicory and lucerne were collected 2–3 cm above the ground level during spring season 2019 (late October 2019). Chicory and pasture were in the second year of cultivation. During spring, both pastures grow faster and produce greater dry matter yield due to warm weather with adequate rainfall, which is perfect conditions for the assimilation of nutrients and other secondary compounds in the vegetative parts. For both species, samples (vegetative parts) were collected from five locations within a particular paddock located at Hamilton Research Station, Vic, Australia and bulked in a ziplock plastic bag, weighing approximately 1000 g in total. The stage of maturity of both forages during collection time were at pre-bloom. Upon collection, on the same day, samples were brought to the University of Melbourne, Parkville 3030 under refrigerated conditions using an esky with ice. Upon arrival, samples were stored at 4 °C until further processing for grinding and chemical extraction.

### 2.2. Chemicals and Reagents

The chemicals used were of analytical purity. Methanol, ethanol, sodium acetate, HCl and glacial acetic acid were procured from Thermo Fisher Scientific Inc. (Waltham, MA, USA). H_2_SO_4_ (98%) was procured from RCI Labscan (Rongmuang, Thailand). Na_2_CO_3_ was purchased from Chem-Supply Pty Ltd (Adelaide, Australia). Folin-Ciocalteu reagent, 2,4,6-tripyridyl-s-triazine (TPTZ), 2,2-diphenyl-1-picrylhydrazyl (DPPH), 2,2′-azino-bis (3-ethylbenzothiazoline-6-sulphonic acid) (ABTS), quercetin, gallic acid, vanillin, L-ascorbic acid, AlCl_3_.6H_2_O, FeCl_2_ Ferrous sulphate, hydrogen peroxide (H_2_O_2_), 3-hydroxybenzoic acid, Ferrozine, Potassium ferricyanide, Trichloroacetic acid and FeCl_3_ were procured from Sigma-Aldrich (Castle Hill, New South Wales, Australia).

### 2.3. Samples Preparation

Within a week of collection, bulk samples (chicory and lucerne) were crushed using a mortar and pestle to make a paste-like consistency, in order to facilitate the extraction of polyphenol compounds and stored at −20 °C for further analysis.

### 2.4. Extraction of Phenolics

Samples pastes were extracted with 80% ethanol, and then the sample-ethanol mixtures were homogenised. Homogenised mixtures were incubated in a ZWYR-240 shaker incubator (Labwit, Ashwood, VIC, Australia). Afterwards, centrifugation was performed on a Hettich Rotina 380R centrifuge machine (Tuttlingen, Germany) for 20 min at 5000 rpm (4 °C). The supernatants were collected and filtered through 0.22 µm syringe filter (PTFE membrane) and stored at −20 °C for the characterisation and quantification of polyphenols.

### 2.5. Antioxidant Assays

Antioxidant activities were determined by previously reported methods [20] using 96-well plates. Absorbance was recorded on Multiskan^®^ Go microplate photometer (Thermo Fisher Scientific, Waltham, MA, USA) and standard curves with R^2^ ≥ 0.99 were constructed with standard solutions. Results were reported on dry weight basis.

#### 2.5.1. Total Phenolics Content (TPC)

Total phenolic content was determined by the Folin and Ciocalteu’s method [21] with slight modifications using a 96-well plate. Sample (25 µL) was mixed with 25 µL Folin’s Reagent (diluted to 1:3 with water) and allowed to incubate at 25 °C for 5 min. Finally, water (200 µL) and 10% (*w*/*w*) Na_2_CO_3_ solution (25 µL) were added. This mixture was incubated for 60 min at 25 °C. Absorbance was recorded at 765 nm with a microplate reader. Measurements were made in triplicate, and quantification was done by constructing a standard curve (0–200 µg/mL gallic acid).

#### 2.5.2. Total Flavonoids Content (TFC)

Total flavonoid content for all sample extracts was measured by the AlCl_3_ colorimetric method [22] with slight modifications using a 96-well plate. 80 µL sample extract was mixed with 80 µL of 2% AlCl_3_ solution. 120 µL of aqueous solution of sodium acetate (50 g/L) was added. This mixture was incubated for 150 min at 25 °C. Absorbance was taken at 440 nm. Measurements for all samples were made in triplicate, and quantification was done by constructing a standard curve (0–50 µg/mL quercetin).

#### 2.5.3. Total Tannin Contents (TTC)

The total tannin content of samples was measured through a colorimetric method [23] with minor changes. Sample extracts (25 µL) was mixed with 4% vanillin solution (150 µL). 25 µL of H_2_SO_4_ solution (32%) was added. This mixture was incubated at 25 °C for 15 min. Absorbance was taken at 500 nm. Measurements were made in triplicate and quantification were done by constructing a standard curve (0–1000 µg/mL catechin solution).

#### 2.5.4. 2,2-Diphenyl-1-picrylhydrazyl (DPPH) Assay

The radical scavenging potential of the samples was estimated by using a previously reported method [24] with slight modifications. 260 µL 0.1 M DPPH solution was mixed with 40 µL sample extract, and allowed to incubate at 25 °C for 30 min. Absorbance was taken at 517 nm. Measurements for all samples were made in triplicate and quantification was done by constructing a standard curve (0–50 µg/mL ascorbic acid).

#### 2.5.5. Ferric Reducing Antioxidant Power (FRAP) Assay

Ferric reducing potential was estimated by a previously used method [24] with modifications in a 96-well plate. 300 mM acetate buffer, 20 mM ferric chloride and 10 mM TPTZ were mixed in 10:1:1 (*v*/*v*/*v*) ratio to prepare FRAP reagent. 280 µL of FRAP reagent was added to 20 µL of sample extract and incubated for 10 min at 37 °C. Absorbance was recorded at 593 nm. Measurements were made in triplicate and quantification was done by constructing a standard curve (0–50 µg/mL ascorbic acid).

#### 2.5.6. 2,2-Azino-bis-3-ethylbenzothiazoline-6-sulfonic Acid (ABTS) Radical Scavenging Assay

The potential of samples to scavenge ABTS radical was determined by ABTS^+^ radical cation de-colorisation method [24] with slight modifications. 7 mM ABTS solution was mixed with 140 mM K_2_S_2_O_8_ solution, and allowed to incubate in the dark for 16 h for the generation of ABTS^+^ ions in the solution. The absorbance of this solution was adjusted to 0.70 ± 0.02 by dilution with ethanol. After this, 10 µL of sample extracts were mixed with ABTS^+^ solution (290 µL), and allowed to incubate at 25 °C for 6 min. Absorbance was taken at 734 nm. Measurements for all samples were made in triplicate, and quantification was done by constructing a standard curve (0–150 µg/mL ascorbic acid).

#### 2.5.7. Hydroxyl (OH^−^) Radical Scavenging Activity Assay

The hydroxyl (OH^−^) radical scavenging potential of samples was determined by translating the method of Pavithra and Vadivukkarasi [25] to a 96-well plate method. 50 µL of sample was mixed with 50 µL of 6 mM Ferrous sulphate solution and 50 µL of 6 mM hydrogen peroxide (H_2_O_2_) solution in a 96-well plate. The mixture was incubated for 10 min at 25 °C. After incubation, 50 µL of 6 mM 3-hydroxybenzoic acid solution was added. Absorbance was recorded at 510 nm. Measurements for all samples were made in triplicate and quantification was done by constructing a standard curve (0–300 µg/mL ascorbic acid).

#### 2.5.8. Chelating Ability of Ferrous Ion (Fe^2+^)

The chelating ability of samples was determined by translating the method of Pavithra and Vadivukkarasi [25] to a 96-well plate method. 100 µL of sample was mixed with 80 µL of 2 mM ferrous chloride solution in a 96-well plate. 70 µL of 5 mM Ferrozine solution was added and mixed well. The mixture was allowed to stand for 10 min at 25 °C. Absorbance was taken at 562 nm. Measurements for all samples were made in triplicate, and quantification was done by constructing a standard curve (0–10 µg/mL EDTA solution).

#### 2.5.9. Reducing Power Assay (RPA)

The reducing power of the samples was determined by translating the method of Pavithra and Vadivukkarasi [25] to a 96-well plate method. 10 µL of sample, 25 µL of 0.2 M phosphate buffer (pH 6.6) and 25 µL of Potassium ferricyanide solution (1% *w*/*v*) were added sequentially in a 96-well plate, and incubated for 20 min at 25 °C. Following incubation, 25 µL of TCA solution (10% *w*/*v*) was added. After this, 85 µL of water and 8.5 µL of Iron (III) chloride solution (0.1% *w*/*v*) and incubated for 15 min at 25 °C. Absorbance was recorded at 750 nm. Measurements for all samples were made in triplicate, and quantification was done by constructing a standard curve (0–150 µg/mL ascorbic acid).

### 2.6. Profiling of Polyphenols by LC-ESI/QTOF-MS

Profiling of polyphenols from samples was achieved by an already published method [20] by using HPLC system (Agilent 1200 series) coupled to Agilent 6520 Accurate-Mass Q-TOF LC/MS (Agilent Technologies, Santa Clara, CA, USA) with an electrospray ionisation source (ESI). Separation of compounds was performed with RP-80A Column (250 mm × 4.6 nm, 4 µm). Mobile phases (A) and (B) were composed of water/acetic acid solution (98:2 *v*/*v*) and water/acetonitrile/acetic acid (50:49.5:0.5, *v*/*v*/*v*) respectively and elution program was set as: 0–20 min linear gradient from 90% to 75% eluent A; 20–30 min linear gradient from 75% to 65% eluent A; 30–40 min linear gradient from 65% to 60% eluent A; 40–70 min linear gradient from 60% to 45% eluent A; 70–75 min linear gradient from 45% to 20% eluent A; 75–77 min linear gradient from 20% to 0% eluent A; 77–79 min linear gradient from 0% eluent A; 82–85 min linear gradient from 0% to 90% eluent A. The flow rate was maintained at 0.8 mL/min. Sample injection volume was 6 µL. Peaks were identified in negative and positive ionisation modes. Mass spectra were obtained in the *m*/*z* range of 50–1300. Data were analyzed on an Agilent LC/MS/QTOF Mass Hunter Data Acquisition Software (Version B.03.01).

### 2.7. Quantification of Polyphenols

Quantification of polyphenols was performed by the previously applied method [26] on a HPLC system (Waters Alliance 2690, Chromatograph Separation Module) connected to photodiode array detector (Model 2998, Waters). The column and conditions of analysis were kept same as applied in the LC- MS analysis, with 20µL injection volume of samples. Peak identification was performed at 280 nm, 320 nm and 370 nm. Data analysis was achieved on Empower Software (2010) (Shimadzu Scientific Instruments, Sydney, NSW, Australia). Each polyphenol was quantified by preparing standard curve (R^2^ ≥ 0.98) with different concentrations of external standards, which include Cinnamic acid (50–250 µg/mL), Gallic acid (62.5–500 µg/mL), Chicoric acid (12.5–200 µg/mL), 2-hydroxybenzoic acid (25–200 µg/mL), *m*-Coumaric acid (12.5–200 µg/mL), *p*-hydroxybenzoic acid (3.125–50 µg/mL), Isorhamnetin (12.5–200 µg/mL), Quercetin 3-rhamnoside (50–250 µg/mL) and Epicatechin gallate (31.25–250 µg/mL).

### 2.8. Statistics Analysis

Results were reported as mean ± standard deviation of the values of three independent analyses. The students’ *t*-test was performed using Microsoft Excel software (Microsoft Corporation, Redmond, WA, USA) to test statistical significance by comparing the means (significant difference at *p* ≤ 0.05). 

## 3. Results and Discussion

### 3.1. Polyphenols Estimation from Chicory and Lucerne Extracts (TPC, TFC and TTC)

Chicory and lucerne are rich in polyphenols. The polyphenol content of chicory and lucerne was determined as TPC, TFC and TTC. The results showed that polyphenolic contents varied considerably in chicory and lucerne (Table 1). Lucerne showed a significantly higher TPC (0.71 ± 0.01 mg GAE/g) and TTC (1.32 ± 0.08 mg CE/g) as compared to chicory, with TPC and TTC values of 0.44 ± 0.04 mg GAE/g and 0.84 ± 0.03 mg CE/g respectively. The TPC of lucerne was determined previously by Zagórska-Dziok et al., [27] in the range of 3.52 mg GAE/g to 73.5 mg GAE/g using different concentrations of water-glycerine extracts of lucerne. Our values for TPC of lucerne were lower than the already reported values. The difference could be explained by the use of different solvents for extraction and the methods applied for determination. No significant difference was observed in total flavonoid content (0.07 ± 0.01 mg QE/g in chicory and 0.07 ± 0.01 mg QE/g in lucerne) in both samples (*p* ≤ 0.05).

Kaur and coworkers [28] also determined TPC of chicory extracts, and were found in the range of 23.4 to 62.5 mg GAE/100 g dry weight (0.234 to 0.625 mg GAE/g dry weight) using different solvents for extraction. Our results for TPC of chicory (0.44 ± 0.04 mg GAE/g dry weight) is within the range already reported by Kaur and coworkers.

### 3.2. Antioxidant Activities of Chicory and Lucerne Extracts as Determined by DPPH, FRAP, ABTS, RPA, OH^−^ Radical Scavenging Ability, Chelating Ability of Fe^2+^

Chicory and lucerne contain phenolic acids and flavonoids that have high antioxidant capacity [29]. The antioxidant activity of chicory and lucerne was determined by DPPH, FRAP, ABTS, OH^−^ radical scavenging ability, chelating ability of Fe^2+^ and reducing power assays (Table 2). These assays are commonly applied for the determination of antioxidant potential of plant extracts [30]. Lucerne showed significantly higher values (*p* ≥ 0.05) of ABTS (1.28 ± 0.02 mg AAE/g), OH^−^ radical scavenging ability (11.29 ± 0.25 mg AAE/g), chelating ability of Fe^2+^ (0.21 ± 0.01 mg EDTAE/g) and reducing power assay (0.59 ± 0.02 mg AAE/g) as compared to chicory that showed 0.27 ± 0.01 mg AAE/g, 8.04 ± 0.33, 0.07 ± 0.01 mg EDTAE/g and 0.34 ± 0.01 mg AAE/g for ABTS, OH^−^ radical scavenging ability, chelating ability of Fe^2+^ and reducing power assay respectively (Table 2).

No significant difference was observed for DPPH and FRAP values for chicory and lucerne. DPPH and FRAP values of chicory are 0.12 ± 0.01 mg AAE/g and 0.01 ± 0.01 mg AAE/g respectively. Meanwhile, lucerne showed DPPH and FRAP values as 0.13 ± 0.01 mg AAE/g and 0.02 ± 0.01 mg AAE/g, respectively.

### 3.3. Polyphenols Profile of Chicory and Lucerne

Profiling of polyphenols from chicory and lucerne were performed by verifying *m*/*z* value from mass spectra in positive ([M + H]^+^) and negatiM+He ([M − H]^−^) ionisation modes and compounds with mass error less than 10 ppm were selected for the verification of *m*/*z* for characterisation using the personal compound database library. 80 polyphenols were identified in chicory and lucerne extracts, with 14 phenolic acids, 52 flavonoids, three lignans, one stilbene and 10 other polyphenols (Table 3). Higher diversity of polyphenols was found in lucerne extract with a total of 56 compounds (Table S2—Supplementary materials), as compared to the chicory extract in which 29 polyphenols (Table S1—Supplementary materials) were identified. Flavonoids and phe-nolic acids were the main polyphenol subgroups in both plant extracts. Stilbenes was only identified in the chicory extract (Supplementary materials—Figure S1).

#### 3.3.1. Phenolic Acids

A total of fourteen phenolic acids belonging to three different subclasses (hydroxybenzoic acids, hydroxycinnamic acids and hydroxyphenylpropanoic acids) were tentatively identified in chicory and lucerne extracts. Hydroxycinnamic acids and hydroxybenzoic acids were the dominant subgroups of phenolic acids, with seven and five compounds, respectively. Only two compounds belonging to hydroxyphenylpropanoic acids were tentatively identified.

##### Hydroxybenzoic Acid Derivatives

Five compounds (Compound **1**, **2**, **3**, **4** & **5**) were tentatively identified as hydroxybenzoic acids in both samples. Compounds (**1** & **2**) were tentatively identified in lucerne extract in positive ionisation mode at *m*/*z* 155.0336 and *m*/*z* 139.0389 and designated as 2,3-Dihydroxybenzoic acid (C_7_H_6_O_4_) and 2-hydroxybenzoic acid (C_7_H_6_O_3_), respectively. Compound (**3**) was tentatively identified in chicory extract in positive ionisation mode at *m*/*z* 301.0934 and designated as 4-hydroxybenzoic acid 4-O-glucoside (C_13_H_16_O_8_). Compounds (**4** & **5**) were tentatively identified in the chicory extract in the negative ionisation mode at *m*/*z* 331.0652 and 463.0546, and were designated as Gallic acid 4-O-glucoside (C_13_H_16_O_10_) and Ellagic acid glucoside (C_20_H_16_O_13_), respectively.

##### Hydroxycinnamic Acid Derivatives

Hydroxycinnamic acid were the predominant phenolic acids in chicory and lucerne [31,32]. Compounds (**6**, **7**, **8**, **9**, **10**, **11** & **12**) were identified as hydroxycinnamic acid derivatives. Compound (**12**) was identified in negative ionisation mode in both chicory and lucerne extracts at *m*/*z* 473.0764 and *m*/*z* 473.0696, respectively, and was designated as chicoric acid (C_22_H_18_O_12_). Chicoric acid has already been reported in methanolic extracts of chicory [17,33]. Compounds (**6**, **9** & **11**) were identified only in the lucerne extract in positive ionisation mode at *m*/*z* 165.0548, 399.1288 and 517.1319, and were designated as *m*-Coumaric acid (C_9_H_8_O_3_), 3-Sinapoylquinic acid (C_18_H_22_O_10_) and 1,5-Dicaffeoylquinic acid (C_25_H_24_O_12_), respectively. *m*-Coumaric acid was also previously identified in lucerne [34]. Compound (**8**) was identified in the lucerne extract in negative ionisation mode at *m*/*z* 616.1062 and designated as 2-S-Glutathionyl caftaric acid (C_23_H_27_N_3_O_15_S). However, compounds (**7** & **10**) were identified in chicory extract in positive ionisation mode at *m*/*z* 149.0585 and 355.0999 and were designated as Cinnamic acid (C_9_H_8_O_2_) and 3-Caffeoylquinic acid, respectively (C_16_H_18_O_9_). 3-Caffeoylquinic acid has also previously been identified in chicory [17]. Out of the seven hydroxycinnamic acids identified in this study, three are in the form of quinic acid derivatives. This agrees with a previous finding that hydroxycinnamic acids mainly exist in conjugated form, such as quinic acid [35].

##### Hydroxyphenylpropanoic Acids

Two hydroxyphenylpropanoic acids (compound **13** & **14**) were identified in negative ionisation mode ([M − H]^−^). Compound (**13**) was identified in both chicory and lucerne extracts in negative ionisation mode at *m*/*z* 357.0847 and 357.0819 respectively and designated as Dihydrocaffeic acid 3-*O*-glucuronide (C_15_H_18_O_10_). However, compound (**14**) was only identified in lucerne extract in negative ionisation mode at *m*/*z* 275.0218, and designated as Dihydroferulic acid 4-sulfate (C_10_H_12_O_7_S).

#### 3.3.2. Flavonoids and Their Derivatives

Higher diversity of flavonoids derivatives was found among the phenolic compounds identified in chicory and lucerne extracts. A total of 52 flavonoids belonging to seven subgroups were identified in this study.

##### Anthocyanins Derivatives

Anthocyanins provide protection to arteries and endothelial tissues, inhibit platelet aggregation and reduce the risk of heart diseases [17,36,37,38]. Chicory and lucerne have been reported to contain different anthocyanin derivatives. The anthocyanin derivatives found in chicory are of special interest due to their beneficial effects on visual capacity, brain cognitive function, obesity and cancer prevention [39,40]. Five anthocyanins (compounds **15**, **16**, **17**, **18** & **19**) were detected in this study. Out of the five anthocyanins, one compound (**18**) was putatively identified in negative ionisation mode at *m*/*z* 640.145 and 640.1413 in both chicory and lucerne extracts, and was designated as Delphinidin 3-*O*-feruloyl-glucoside (C_31_H_29_O_15_). Compounds (**15** & **19**) were putatively identified in negative ionisation mode in lucerne extract at *m*/*z* 300.0654 and 740.2187 and designated as Peonidin (C_16_H_13_O_6_) and Pelargonidin 3-*O*-glucosyl-rutinoside (C_33_H_41_O_19_), respectively. Compound (**16**) was tentatively identified in chicory extract in negative ionisation mode at *m*/*z* 696.1516 and designated as Cyanidin 3-*O*-(6″-malonyl-3″-glucosyl-glucoside) with molecular formula C_30_H_33_O_19_. Compound (**17**) was identified at *m*/*z* 626.166 in positive ionisation mode in chicory extract, and designated as Petunidin 3-*O*-(6″-*p*-coumaroyl-glucoside) with the molecular formula C_31_H_29_O_14_.

##### Dihydrochalcones

Only one dihydrochalcone compound (compound **20**) with molecular formula C_21_H_22_O_12_ was detected in this study in both chicory and lucerne extracts. It was designated as Dihydromyricetin 3-*O*-rhamnoside. Dihydromyricetin 3-O-rhamnoside was putatively identified in the chicory extract in negative ionisation mode at *m*/*z* 465.1041, while it was identified in positive ionisation mode at *m*/*z* 467.1162 in the lucerne extract.

##### Flavanols Derivatives

A total of four flavanols (compounds **21**, **22**, **23** & **24**) were detected in this study. Two flavanols (compounds **21** & **22**) were tentatively identified in the lucerne extract in positive ionisation mode. Compound (**21**) identified at *m*/*z* 321.0986 with a formula of C_16_H_16_O_7_ was designated as 4′-*O*-Methylepigallocatechin, while compound (**22**) was identified at *m*/*z* 473.1049 and designated as 4″-*O*-Methylepigallocatechin 3-*O*-gallate (C_23_H_20_O_11_). The other two flavanols (compounds **23** & **24**) were tentatively identified in chicory extract in negative ionisation mode. Compound (**23**) identified at *m*/*z* 481.1007 with the formula C_21_H_22_O_13_ was designated as (-)-Epigallocatechin 3′-*O*-glucuronide, while compound (**24**) was identified at *m*/*z* 479.1214 with the molecular formula C_22_H_24_O_12_, and was designated as 3′-*O*-Methyl-(-)-epicatechin 7-*O*-glucuronide.

##### Flavanones Derivatives

Five flavanones (compounds **25**, **26**, **27**, **28** & **29**) were detected in the present study. Of these, four flavanones (compounds **25**, **26**, **27** & **28**) were detected in the lucerne extract, while compound (**29**) was detected in the chicory extract. Compound (**29**), with a precursor ion at *m*/*z* 477.107 in negative ionisation mode with a molecular formula of C_22_H_22_O_12_ was tentatively identified as Hesperetin 3′-*O*-glucuronide. Compound (**25**), with molecular formula C_27_H_32_O_14_, was detected in negative ionisation mode at *m*/*z* 579.1719 and designated as Narirutin. Compounds (**26**, **27** & **28**) were detected at *m*/*z* 597.1853, 655.1523 and 435.1266, respectively, in positive ionisation mode. Compound (**26**), with the molecular formula C_27_H_32_O_15_, was designated as Neoeriocitrin, while compounds (**27** & **28**) with the molecular formula C_28_H_30_O_18_ and C_21_H_22_O_10_ were designated as Hesperetin 3′,7-*O*-diglucuronide and Naringenin 7-*O*-glucoside.

##### Flavones Derivatives

Seven flavones (compounds **30**, **31**, **32**, **33**, **34**, **35** & **36**) were detected. Compound (**36**) was detected in both the chicory and lucerne extracts. It was detected in chicory extract in negative ionisation mode at *m*/*z* 431.0979 and in lucerne extract in positive ionisation mode at *m*/*z* 433.1126, and tentatively identified as Apigenin 6-C-glucoside (C_21_H_20_O_10_). Compound (**33**) was only detected in chicory extract in positive ionisation mode at *m*/*z* 463.1231, and was designated as Chrysoeriol 7-*O*-glucoside (C_22_H_22_O_11_). Compounds (**30**, **31**, **32**, **34** & **35**) were only detected in the lucerne extract in positive ionisation mode at *m*/*z* 639.1192, 449.1073, 447.0925, 549.1236 and 255.0647, respectively. These compounds (**30**, **31**, **32**, **34** & **35**) with molecular formulae C_27_H_26_O_18_, C_21_H_20_O_11_, C_21_H_18_O_11_, C_25_H_24_O_14_ and C_15_H_10_O_4_ were tentatively identified as Luteolin 7-*O*-diglucuronide, 6-Hydroxyluteolin 7-*O*-rhamnoside, Apigenin 7-*O*-glucuronide, Chrysoeriol 7-*O*-(6″-malonyl-glucoside) and 7,4′-Dihydroxyflavone.

##### Flavonols Derivatives

Eleven flavonols (compounds **37**, **38**, **39**, **40**, **41**, **42**, **43**, **44**, **45**, **46** & **47**) were detected in samples in this study. Out of the eleven flavonols, seven (compounds **38**, **39**, **40**, **44**, **45**, **46** & **47**) were detected in chicory extract and four (compounds **37**, **41**, **42** & **43**) were detected in lucerne extract. Compounds (**37**, **41** & **42**) were putatively identified in negative ionisation mode in lucerne extract at *m*/*z* 625.1404, 801.2084 and 533.0928 and designated as Myricetin 3-*O*-rutinoside (C_27_H_30_O_17_), Spinacetin 3-*O*-glucosyl-(1->6)-[apiosyl(1->2)]-glucoside (C_34_H_42_O_22_) and 5,4′-Dihydroxy-3,3′-dimethoxy-6:7-methylenedioxyflavone 4′-*O*-glucuronide (C_24_H_22_O_14_), respectively. Meanwhile, compound (**43**) was tentatively identified at *m*/*z* 331.0812 in positive ionisation mode in the lucerne extract, with molecular formula C_17_H_14_O_7_, and designated as 3,7-Dimethylquercetin.

Compounds (**38**, **39**, **40** & **45**) were putatively identified in positive ionisation mode in chicory extract at *m*/*z* 637.1777, 403.1367, 317.0666 and 625.176 having molecular formulae C_29_H_32_O_16_, C_21_H_22_O_8_, C_16_H_12_O_7_ and C_28_H_32_O_16,_ respectively. These compounds (**38**, **39**, **40** & **45**) were tentatively identified as Kaempferol 3-*O*-(6″-acetyl-galactoside) 7-*O*-rhamnoside, 3-Methoxysinensetin, Isorhamnetin and Isorhamnetin 3-*O*-glucoside 7-*O*-rhamnoside respectively. Isorhamnetin derivatives have also been previously identified in chicory [17]. Compounds (**44**, **46** & **47**) were detected in negative ionisation mode in chicory extract at *m*/*z* 595.1286, 449.0757 and 491.0856 with molecular formulae C_26_H_28_O_16_, C_20_H_18_O_12_ and C_22_H_20_O_13_, and were tentatively identified as Quercetin 3-*O*-glucosyl-xyloside, Myricetin 3-*O*-arabinoside and Isorhamnetin 3-*O*-glucuronide, respectively.

##### Isoflavonoids Derivatives

A total of nineteen isoflavonoids were detected in the present work. Of these, seventeen compounds (**49**, **50**, **52**, **53**, **54**, **55**, **56**, **57**, **58**, **59**, **60**, **61**, **62**, **63**, **64**, **65** & **66**) were detected in lucerne extract and two compounds (**48** & **51**) were detected in chicory extract. Compounds (**48** & **51**) detected in negative ionisation mode at *m*/*z* 487.1232 and 285.0758 with molecular formulae C_24_H_24_O_11_ and C_16_H_14_O_5_ were tentatively identified as 6″-*O*-Acetylglycitin and Dihydrobiochanin A, respectively.

Compounds (**49**, **52**, **53**, **54**, **55**, **58**, **59**, **60**, **61**, **62**, **63**, **64**, **65** & **66**) traced in positive ionisation mode in lucerne extract at *m*/*z* 289.0704, 417.1195, 431.0968, 623.1238, 491.1189, 519.115, 257.0801, 315.0855, 285.075, 273.0746, 533.1301, 271.0594, 301.0699 and 269.0788 with molecular formulae C_15_H_12_O_6_, C_21_H_20_O_9_, C_21_H_18_O_10_, C_27_H_26_O_17_, C_23_H_22_O_12_, C_24_H_22_O_13_, C_15_H_12_O_4_, C_17_H_14_O_6_, C_16_H_12_O_5_, C_15_H_12_O_5_, C_25_H_24_O_13_, C_15_H_10_O_5_, C_16_H_12_O_6_ and C_16_H_12_O_4_ were tentatively identified as 3′,4′,5,7-Tetrahydroxyisoflavanone, Puerarin, Daidzein 4′-*O*-glucuronide, Genistein 4′,7-*O*-diglucuronide, Irisolidone 7-*O*-glucuronide, 6″-*O*-Malonylgenistin, 2-Dehydro-*O*-desmethylangolensin, 2′,7-Dihydroxy-4′,5′-dimethoxyisoflavone, 2′-Hydroxyformononetin, 3′,4′,7-Trihydroxyisoflavanone, 6″-*O*-Malonylglycitin, 3′-Hydroxydaidzein, 3′-Hydroxymelanettin and Dalbergin, respectively. Meanwhile, compounds (**50**, **56** & **57**) detected in negative ionisation mode in lucerne extract at *m*/*z* 285.042, 379.0147 and 501.1035 having molecular formulae C_15_H_10_O_6_, C_16_H_12_O_9_S and C_24_H_22_O_12_ were tentatively identified as 3′-Hydroxygenistein, Tectorigenin 7-sulfate and 6″-*O*-Malonyldaidzin, respectively.

#### 3.3.3. Lignans and Stilbenes

Three lignans (compounds **67**, **68** & **69**) and one stilbene (compound **70**) were detected in this work. Compound (**67**) traced in positive ionisation mode at *m*/*z* 355.1171 with molecular formula C_20_H_18_O_6_ was designated as Sesamin, and compound (**68**) detected in negative ionisation mode at *m*/*z* 387.1457 was tentatively identified as Trachelogenin (C_21_H_24_O_7_) in the lucerne extract only. On the other hand, compound (**69**) was only detected in the chicory extract in negative ionisation mode at *m*/*z* 415.1389, and tentatively characterised as 1-Acetoxypinoresinol (C_22_H_24_O_8_). The only stilbene (compound **70**) was tentatively identified in chicory extract in positive ionisation mode, at *m*/*z* 245.0809, with molecular formula C_14_H_12_O_4_ and designated as Piceatannol.

#### 3.3.4. Other Polyphenols

Four categories of other polyphenols were found in samples in the present study. These categories included one alkylphenols (compound **71**), one hydroxybenzaldehydes (compound **72**), four tyrosols (compounds **73**, **74**, **75** & **76**) and four other polyphenols (compounds **77**, **78**, **79** & **80**). Compounds (**71** & **72**) detected in lucerne extract in positive ionisation mode at *m*/*z* 121.0638 and 123.044 with molecular formulae C_8_H_8_O and C_7_H_6_O_2_ were tentatively identified as 4-Vinylphenol and 4-Hydroxybenzaldehyde, respectively. Among tyrosols, compound (**73**) was detected only in the chicory extract in negative ionisation mode at *m*/*z* 377.1238, and with molecular formula C_19_H_22_O_8_ was tentatively identified as Oleuropein-aglycone. On the other hand, the other three tyrosols (compound **74**, **75** & **76**) were only detected in the lucerne extract. Compound (**74**) traced in positive ionisation mode at *m*/*z* 197.0809 having molecular formula C_10_H_12_O_4_ was tentatively identified as 3,4-DHPEA-AC. Compounds (**75** & **76**) were traced in negative ionisation mode at *m*/*z* 403.1266 and 319.1179 with molecular formulae C_17_H_24_O_11_ and C_17_H_20_O_6_, and were tentatively identified as Oleoside 11-methylester and 3,4-DHPEA-EDA, respectively.

Among other polyphenols, compound (**79**) was only detected in the chicory extract, while compounds (**77**, **78** & **80**) were only detected in the lucerne extract. Compounds (**77** & **78**) traced at *m*/*z* 237.0408 and 491.1007 in negative ionisation mode with molecular formula C_11_H_10_O_6_ and C_26_H_20_O_10_ were designated as Salvianolic acid D and Salvianolic acid C, respectively. Compound (**79**) detected in chicory extract at *m*/*z* 539.1225 in positive ionisation mode with molecular formula C_27_H_22_O_12_ was tentatively identified as Lithospermic acid, while compound (**80**) detected at *m*/*z* 269.0437 in lucerne extract in positive ionisation mode with the molecular formula C_15_H_8_O_5_ was tentatively identified as Coumestrol.

### 3.4. Quantification of Polyphenols through HPLC-PDA

HPLC is a commonly applied technique for the quantification of polyphenols from various types of samples. The phenolic acids and flavonoids have medicinal importance, and are well known for their high antioxidant capabilities. These are the main compounds responsible for the high antioxidant potential of plant extracts. Therefore, we quantified four phenolic acids (Cinnamic acid, Chicoric acid, 2-hydroxybenzoic acid and *m*-Coumaric acid) and one flavonoid (Isorhamnetin) in chicory and lucerne, as these compounds are commonly found polyphenols in chicory and lucerne.

Table 4 shows the data of targeted polyphenolic compounds quantified in chicory and lucerne. Of the nine targeted polyphenols, six compounds belong to the phenolic acids and three are flavonoids. Two compounds (Cinnamic acid and Isorhamnetin) were detected and quantified only in chicory and three compounds (2-hydroxybenzoic acid, *m*-Coumaric acid and *p*-hydroxybenzoic acid) quantified in lucerne only. Two phenolic acids (gallic acid and chicoric acid) were detected and quantified in both chicory and lucerne. The concentration of gallic acid was 38.17 ± 0.03 µg/g DW in chicory and 55.74 ± 0.04 µg/g DW in lucerne. Chicoric acid is the major phenolic acid in chicory, with the highest concentration (1692.33 ± 0.04 µg/g DW) among all phenolic compounds quantified in this study. The concentration of chicoric acid in lucerne was lower (1434.36 ± 0.02 µg/g DW) as compared to chicory. *p*-hydroxybenzoic acid was quantified only in lucerne (11.55 ± 0.02). Cinnamic acid concentration was 115.00 ± 0.01 µg/g DW in chicory. 2-hydroxybenzoic acid and *m*-coumaric acid concentrations in lucerne were 1440.64 ± 0.04 µg/g DW and 2.64 ± 0.01 µg/g DW, respectively. Out of the three detected flavonoids, isorhamnetin was quantified in only the chicory extract at a concentration of 641.80 ± 0.03 µg/g DW. Meanwhile, quercetin 3-rhamnoside and epicatechin gallate were detected and quantified in both chicory and lucerne. Quercetin 3-rhamnoside concentration in lucerne was much higher (187.74 ± 0.05 µg/g DW) as compared to chicory (5.50 ± 0.04 µg/g DW). The concentration of epicatechin gallate was 29.28 ± 0.02 µg/g DW and 62.77 ± 0.03 µg/g DW in chicory and lucerne, respectively.

### 3.5. Relationship of Phenolic Contents and Antioxidant Activities

Polyphenols found in chicory and lucerne contribute significantly to their bioactive potential, owing to their strong antioxidant activities. Considering the role of phenolic contents in antioxidant potential, we investigated the TPC, TFC and TTC. Lucerne showed higher values of TPC, TFC and TTC as compared to chicory (Table 1). The DPPH, ABTS, FRAP, OH^−^ Radical Scavenging Ability, Chelating Ability of Fe^2+^ and RPA were measured to determine the antioxidant potential of chicory and lucerne. Lucerne showed higher values for the all the antioxidant activities corresponding to its high phenolic contents (TPC, TFC and TTC). Therefore, it could be established that the phenolic contents of the plants highly contributed to their antioxidant activities. Results of the study showed that phenolic contents of chicory and lucerne are significant contributors of their antioxidant potential and bioactive properties. However, the antioxidant effects of different phenolic constituents could vary owing to their concentration, synergistic action and antagonistic actions with other chemical moieties present in chicory and lucerne.

## 4. Conclusions

A major finding of this study was that the lucerne has higher level of phenolic compounds (TPC, TFC and TTC) and greater antioxidant potential (DPPH, FRAP, ABTS, Hydroxyl (OH^−^) Radical Scavenging Activity, Chelating Ability of Ferrous Ion (Fe^2+^) and Reducing Power) than chicory. This was supported by the LC-ESI-QTOF/MS analysis, since a higher diversity of polyphenols was observed in the vegetative parts of lucerne (56 compounds) when compared with chicory (29 compounds). Among the polyphenols identified, phenolic acids and flavonoids were the most common polyphenols present in both lucerne and chicory forages. Hence, chicory and lucerne could serve as good sources of antioxidant polyphenols. Moreover, the obtained results could support these plants’ utilisation as ingredients of natural feed additives in animal feeds, functional foods and pharmaceutical formulations. However, further experimental work conducted in animals in vivo is needed to understand the mode of actions of these polyphenols in the body, their inclusion levels in the diet and feeding length that can improve the performance or wellbeing of farm animals and humans.

## Figures and Tables

**Table 1:** Total phenolics content (TPC), total flavonoids content (TFC) and total tannins content (TTC) of chicory and lucerne.

Phenolic Content	Chicory	Lucerne
TPC (mg GAE/g)	0.44 ± 0.04 ^a^	0.71 ± 0.01 ^b^
TFC (mg QE/g)	0.07 ± 0.01 ^a^	0.07 ± 0.01 ^a^
TTC (mg CE/g)	0.84 ± 0.03 ^a^	1.32 ± 0.08 ^b^

Results are reported on a dry weight basis; n = three replicates per sample. The terms mg GAE/g, mg QE/g and mg CE/g for milligrams of gallic acid equivalents, milligrams of quercetin equivalents and milligrams of catechin equivalents, respectively. Within a row, significant difference (*p* ≤ 0.05) is indicated by superscript letters (^a,b^).

**Table 2:** Antioxidant activities of chicory and lucerne extracts.

Antioxidant Activity	Chicory	Lucerne
DPPH (mg AAE/g)	0.12 ± 0.01 ^a^	0.13 ± 0.01 ^a^
ABTS (mg AAE/g)	0.27 ± 0.01 ^a^	1.28 ± 0.02 ^b^
FRAP (mg AAE/g)	0.01 ± 0.01 ^a^	0.02 ± 0.01 ^a^
OH^−^ Radical Scavenging Ability (mg AAE/g)	8.04 ± 0.33 ^a^	11.29 ± 0.25 ^b^
Chelating Ability of Fe^2+^ (mg EDTAE/g)	0.07 ± 0.01 ^a^	0.21 ± 0.01 ^b^
RPA (mg AAE/g)	0.34 ± 0.01 ^a^	0.59 ± 0.02 ^b^

Results are reported on a dry weight basis; n = three replicates per sample. The terms mg AAE/g and mg EDTAE/g stand for milligrams of ascorbic acid equivalents and mg of Ethylenediaminetetraacetic acid. ^a,b^ Denotes *p* ≤ 0.05.

**Table 3:** Phenolic compounds detected and identified in chicory and lucerne by LC-ESI-QTOF-MS.

Sr. No.	Proposed Compounds	Molecular Formula	RT (min)	Mode of Ionisation	Molecular Weight	Theoretical (*m*/*z*)	Observed (*m*/*z*)	Mass Error (ppm)	Samples
	**Phenolic acids**								
	**Hydroxybenzoic acids**								
1	2,3-Dihydroxybenzoic acid	C_7_H_6_O_4_	12.217	[M + H]^+^	154.0266	155.0339	155.0336	−1.94	Lucerne
2	2-Hydroxybenzoic acid	C_7_H_6_O_3_	13.724	[M + H]^+^	138.0317	139.0390	139.0389	−0.72	Lucerne
3	4-Hydroxybenzoic acid 4-*O*-glucoside	C_13_H_16_O_8_	29.701	[M + H]^+^	300.0845	301.0918	301.0934	5.31	Chicory
4	Gallic acid 4-*O*-glucoside	C_13_H_16_O_10_	45.61	[M − H]^−^	332.0743	331.0670	331.0652	−5.44	Chicory
5	Ellagic acid glucoside	C_20_H_16_O_13_	50.398	[M − H]^−^	464.0591	463.0518	463.0546	6.05	Chicory
	**Hydroxycinnamic acids**								
6	*m*-Coumaric acid	C_9_H_8_O_3_	7.811	[M + H]^+^	164.0473	165.0546	165.0548	1.21	Lucerne
7	Cinnamic acid	C_9_H_8_O_2_	12.425	[M + H]^+^	148.0524	149.0597	149.0585	−8.05	Chicory
8	2-S-Glutathionyl caftaric acid	C_23_H_27_N_3_O_15_S	13.954	[M − H]^−^	617.1163	616.1090	616.1062	−4.54	Lucerne
9	3-Sinapoylquinic acid	C_18_H_22_O_10_	18.196	[M + H]^+^	398.1213	399.1286	399.1288	0.50	Lucerne
10	3-Caffeoylquinic acid	C_16_H_18_O_9_	24.781	[M + H]^+^	354.0951	355.1024	355.0999	−7.04	Chicory
11	1,5-Dicaffeoylquinic acid	C_25_H_24_O_12_	68.797	[M + H]^+^	516.1268	517.1341	517.1319	−4.25	Lucerne
12	Chicoric acid	C_22_H_18_O_12_	82.603	[M − H]^−^	474.0798	473.0725	473.0764	8.24	Chicory * & Lucerne
	**Hydroxyphenylpropanoic acids**								
13	Dihydrocaffeic acid 3-*O*-glucuronide	C_15_H_18_O_10_	28.513	[M − H]^−^	358.09	357.0827	357.0847	5.60	Chicory * & Lucerne
14	Dihydroferulic acid 4-sulfate	C_10_H_12_O_7_S	35.54	[M − H]^−^	276.0304	275.0231	275.0218	−4.73	Lucerne
	**Flavonoids**								
	**Anthocyanins**								
15	Peonidin	C_16_H_13_O_6_	24.126	[M − H]^−^	301.0712	300.0639	300.0654	5.00	Lucerne
16	Cyanidin 3-*O*-(6″-malonyl-3″-glucosyl-glucoside)	C_30_H_33_O_19_	28.513	[M − H]^−^	697.1616	696.1543	696.1516	−3.88	Chicory
17	Petunidin 3-*O*-(6″-*p*-coumaroyl-glucoside)	C_31_H_29_O_14_	49.428	[M + H]^+^	625.1557	626.1630	626.166	4.79	Chicory
18	Delphinidin 3-*O*-feruloyl-glucoside	C_31_H_29_O_15_	51.955	[M − H]^−^	641.1506	640.1433	640.145	2.66	Chicory * & Lucerne
19	Pelargonidin 3-*O*-glucosyl-rutinoside	C_33_H_41_O_19_	79.658	[M − H]^−^	741.2242	740.2169	740.2187	2.43	Lucerne
	**Dihydrochalcones**								
20	Dihydromyricetin 3-*O*-rhamnoside	C_21_H_22_O_12_	84.177	[M + H]^+^/[M − H]^−^ *	466.1111	465.1038	465.1041	0.65	Chicory * & Lucerne
	**Flavanols**								
21	4′-*O*-Methylepigallocatechin	C_16_H_16_O_7_	8.142	[M + H]^+^	320.0896	321.0969	321.0986	5.29	Lucerne
22	4″-*O*-Methylepigallocatechin 3-*O*-gallate	C_23_H_20_O_11_	19.124	[M + H]^+^	472.1006	473.1079	473.1049	−6.34	Lucerne
23	(-)-Epigallocatechin 3′-*O*-glucuronide	C_21_H_22_O_13_	66.318	[M − H]^−^	482.106	481.0987	481.1007	4.16	Chicory
24	3′-*O*-Methyl-(-)-epicatechin 7-*O*-glucuronide	C_22_H_24_O_12_	75.38	[M − H]^−^	480.1268	479.1195	479.1214	3.97	Chicory
	**Flavanones**								
25	Narirutin	C_27_H_32_O_14_	12.463	[M − H]^−^	580.1792	579.1719	579.1719	0.00	Lucerne
26	Neoeriocitrin	C_27_H_32_O_15_	15.645	[M + H]^+^	596.1741	597.1814	597.1853	6.53	Lucerne
27	Hesperetin 3′,7-*O*-diglucuronide	C_28_H_30_O_18_	17.136	[M + H]^+^	654.1432	655.1505	655.1523	2.75	Lucerne
28	Naringenin 7-*O*-glucoside	C_21_H_22_O_10_	60.018	[M + H]^+^	434.1213	435.1286	435.1266	−4.60	Lucerne
29	Hesperetin 3′-*O*-glucuronide	C_22_H_22_O_12_	74.519	[M − H]^−^	478.1111	477.1038	477.107	6.71	Chicory
	**Flavones**								
30	Luteolin 7-*O*-diglucuronide	C_27_H_26_O_18_	20.714	[M + H]^+^	638.1119	639.1192	639.1192	0.00	Lucerne
31	6-Hydroxyluteolin 7-*O*-rhamnoside	C_21_H_20_O_11_	40.689	[M + H]^+^	448.1006	449.1079	449.1073	−1.34	Lucerne
32	Apigenin 7-*O*-glucuronide	C_21_H_18_O_11_	43.107	[M + H]^+^	446.0849	447.0922	447.0925	0.67	Lucerne
33	Chrysoeriol 7-*O*-glucoside	C_22_H_22_O_11_	46.728	[M + H]^+^	462.1162	463.1235	463.1231	−0.86	Chicory
34	Chrysoeriol 7-*O*-(6″-malonyl-glucoside)	C_25_H_24_O_14_	60.201	[M + H]^+^	548.1166	549.1239	549.1236	−0.55	Lucerne
35	7,4′-Dihydroxyflavone	C_15_H_10_O_4_	62.139	[M + H]^+^	254.0579	255.0652	255.0647	−1.96	Lucerne
36	Apigenin 6-C-glucoside	C_21_H_20_O_10_	41.948	[M + H]^+^ */[M − H]^−^	432.1056	433.1129	433.1126	−0.69	Chicory & Lucerne *
	**Flavonols**								
37	Myricetin 3-*O*-rutinoside	C_27_H_30_O_17_	8.454	[M − H]^−^	626.1483	625.1410	625.1404	−0.96	Lucerne
38	Kaempferol 3-*O*-(6″-acetyl-galactoside) 7-*O*-rhamnoside	C_29_H_32_O_16_	24.682	[M + H]^+^	636.169	637.1763	637.1777	2.20	Chicory
39	3-Methoxysinensetin	C_21_H_22_O_8_	26.703	[M + H]^+^	402.1315	403.1388	403.1367	−5.21	Chicory
40	Isorhamnetin	C_16_H_12_O_7_	29.717	[M + H]^+^	316.0583	317.0656	317.0666	3.15	Chicory
41	Spinacetin 3-*O*-glucosyl-(1->6)-[apiosyl(1->2)]-glucoside	C_34_H_42_O_22_	32.757	[M − H]^−^	802.2168	801.2095	801.2084	−1.37	Lucerne
42	5,4′-Dihydroxy-3,3′-dimethoxy-6:7-methylenedioxyflavone 4′-*O*-glucuronide	C_24_H_22_O_14_	42.498	[M − H]^−^	534.101	533.0937	533.0928	−1.69	Lucerne
43	3,7-Dimethylquercetin	C_17_H_14_O_7_	44.73	[M + H]^+^	330.074	331.0813	331.0812	−0.30	Lucerne
44	Quercetin 3-*O*-glucosyl-xyloside	C_26_H_28_O_16_	47.797	[M − H]^−^	596.1377	595.1304	595.1286	−3.02	Chicory
45	Isorhamnetin 3-*O*-glucoside 7-*O*-rhamnoside	C_28_H_32_O_16_	49.593	[M + H]^+^	624.169	625.1763	625.176	−0.48	Chicory
46	Myricetin 3-*O*-arabinoside	C_20_H_18_O_12_	50.265	[M − H]^−^	450.0798	449.0725	449.0757	7.13	Chicory
47	Isorhamnetin 3-*O*-glucuronide	C_22_H_20_O_13_	55.351	[M − H]^−^	492.0904	491.0831	491.0856	5.09	Chicory
	**Isoflavonoids**								
48	6″-*O*-Acetylglycitin	C_24_H_24_O_11_	7.838	[M − H]^−^	488.1319	487.1246	487.1232	−2.87	Chicory
49	3′,4′,5,7-Tetrahydroxyisoflavanone	C_15_H_12_O_6_	19.124	[M + H]^+^	288.0634	289.0707	289.0704	−1.04	Lucerne
50	3′-Hydroxygenistein	C_15_H_10_O_6_	20.034	[M − H]^−^	286.0477	285.0404	285.042	5.61	Lucerne
51	Dihydrobiochanin A	C_16_H_14_O_5_	20.594	[M − H]^−^	286.0841	285.0768	285.0758	−3.51	Chicory
52	Puerarin	C_21_H_20_O_9_	24.341	[M + H]^+^	416.1107	417.1180	417.1195	3.60	Lucerne
53	Daidzein 4′-*O*-glucuronide	C_21_H_18_O_10_	24.556	[M + H]^+^	430.09	431.0973	431.0968	−1.16	Lucerne
54	Genistein 4′,7-*O*-diglucuronide	C_27_H_26_O_17_	25.186	[M + H]^+^	622.117	623.1243	623.1238	−0.80	Lucerne
55	Irisolidone 7-*O*-glucuronide	C_23_H_22_O_12_	26.345	[M + H]^+^	490.1111	491.1184	491.1189	1.02	Lucerne
56	Tectorigenin 7-sulfate	C_16_H_12_O_9_S	31.332	[M − H]^−^	380.0202	379.0129	379.0147	4.75	Lucerne
57	6″-*O*-Malonyldaidzin	C_24_H_22_O_12_	35.872	[M − H]^−^	502.1111	501.1038	501.1035	−0.60	Lucerne
58	6″-*O*-Malonylgenistin	C_24_H_22_O_13_	58.677	[M + H]^+^	518.106	519.1133	519.115	3.27	Lucerne
59	2-Dehydro-*O*-desmethylangolensin	C_15_H_12_O_4_	61.691	[M + H]^+^	256.0736	257.0809	257.0801	−3.11	Lucerne
60	2′,7-Dihydroxy-4′,5′-dimethoxyisoflavone	C_17_H_14_O_6_	64.06	[M + H]^+^	314.079	315.0863	315.0855	−2.54	Lucerne
61	2′-Hydroxyformononetin	C_16_H_12_O_5_	67.008	[M + H]^+^	284.0685	285.0758	285.075	−2.81	Lucerne
62	3′,4′,7-Trihydroxyisoflavanone	C_15_H_12_O_5_	81.004	[M + H]^+^	272.0685	273.0758	273.0746	−4.39	Lucerne
63	6″-*O*-Malonylglycitin	C_25_H_24_O_13_	82.926	[M + H]^+^	532.1217	533.1290	533.1301	2.06	Lucerne
64	3′-Hydroxydaidzein	C_15_H_10_O_5_	83.472	[M + H]^+^	270.0528	271.0601	271.0594	−2.58	Lucerne
65	3′-Hydroxymelanettin	C_16_H_12_O_6_	84.251	[M + H]^+^	300.0634	301.0707	301.0699	−2.66	Lucerne
66	Dalbergin	C_16_H_12_O_4_	87.066	[M + H]^+^	268.0736	269.0809	269.0788	−7.80	Lucerne
	**Lignans**								
67	Sesamin	C_20_H_18_O_6_	22.138	[M + H]^+^	354.1103	355.1176	355.1171	−1.41	Lucerne
68	Trachelogenin	C_21_H_24_O_7_	35.093	[M − H]^−^	388.1522	387.1449	387.1457	2.07	Lucerne
69	1-Acetoxypinoresinol	C_22_H_24_O_8_	45.196	[M − H]^−^	416.1471	415.1398	415.1389	−2.17	Chicory
	**Stilbenes**								
70	Piceatannol	C_14_H_12_O_4_	7.44	[M + H]^+^	244.0736	245.0809	245.0821	4.90	Chicory
	**Other polyphenols**								
	**Alkylphenols**								
71	4-Vinylphenol	C_8_H_8_O	7.894	[M + H]^+^	120.0575	121.0648	121.0638	−8.26	Lucerne
	**Hydroxybenzaldehydes**								
72	4-Hydroxybenzaldehyde	C_7_H_6_O_2_	31.761	[M + H]^+^	122.0368	123.0441	123.044	−0.81	Lucerne
	**Tyrosols**								
73	Oleuropein-aglycone	C_19_H_22_O_8_	7.954	[M − H]^−^	378.1315	377.1242	377.1238	−1.06	Chicory
74	3,4-DHPEA-AC	C_10_H_12_O_4_	20.796	[M + H]^+^	196.0736	197.0809	197.0809	0.00	Lucerne
75	Oleoside 11-methylester	C_17_H_24_O_11_	64.317	[M − H]^−^	404.1319	403.1246	403.1266	4.96	Lucerne
76	3,4-DHPEA-EDA	C_17_H_20_O_6_	67.034	[M − H]^−^	320.126	319.1187	319.1179	−2.51	Lucerne
	**Other polyphenols**								
77	Salvianolic acid D	C_11_H_10_O_6_	6.085	[M − H]^−^	238.0477	237.0404	237.0408	1.69	Lucerne
78	Salvianolic acid C	C_26_H_20_O_10_	21.26	[M − H]^−^	492.1056	491.0983	491.1007	4.89	Lucerne
79	Lithospermic acid	C_27_H_22_O_12_	24.616	[M + H]^+^	538.1111	539.1184	539.1225	7.61	Chicory
80	Coumestrol	C_15_H_8_O_5_	83.986	[M + H]^+^	268.0372	269.0445	269.0437	−2.97	Lucerne

* = compound detected in both chicory and lucerne, data presented only with asterisk.

**Table 4:** Quantification of targeted polyphenols in chicory and lucerne by HPLC-PDA analysis.

No.	Compound Name	RT	Chicory (µg/g DW)	Lucerne (µg/g DW)	Polyphenol Class
1	Gallic acid	5.249	38.17 ± 0.03	55.74 ± 0.04	Phenolic acids
2	Cinnamic acid	12.871	115.00 ± 0.01	-	Phenolic acids
3	2-hydroxybenzoic acid	16.427	-	1440.64 ± 0.04	Phenolic acids
4	*p*-hydroxybenzoic acid	17.129		11.55 ± 0.02	Phenolic acids
5	*m*-Coumaric acid	27.598	-	2.64 ± 0.01	Phenolic acids
6	Isorhamnetin	29.872	641.80 ± 0.03	-	Flavonoids
7	Epicatechin gallate	34.303	29.28 ± 0.02	62.77 ± 0.03	Flavonoids
8	Quercetin 3-rhamnoside	38.477	5.50 ± 0.04	187.74 ± 0.05	Flavonoids
9	Chicoric acid	82.402	1692.33 ± 0.04	1434.36 ± 0.02	Phenolic acids

The terms RT and DW stands for retention time and dry weight.

## Data Availability

The data presented in this study is available in supplementary material.

## Supplementary Materials

The following are available online at https://www.mdpi.com/article/10.3390/antiox10060932/s1. Table S1. Phenolic compounds detected and tentatively characterised in chicory by LC-ESI-QTOF/MS in both positive and negative ionisation modes. Table S2. Phenolic compounds detected and tentatively characterised in lucerne by LC-ESI-QTOF/MS in both positive and negative ionisation modes. Figure S1: LC-ESI-QTOF/MS basic peak chromatographs (BPC) for characterisation of phenolic compounds of chicory and lucerne; (a) Base Peak Chromatogram (BPC) of chicory in negative ionisation mode; (b) TIC of chicory in positive ionisation mode; (c) BPC of lucerne in negative ionisation mode; (d) BPC of lucerne in positive ionisation mode; (e) A chromatograph of chicoric acid (Compound **12** Chicory, Table 3), Retention time (RT = 82.603) in the negative mode of ionisation (ESI-/[M-H]-); (f) Mass spectra of chicoric acid showing *m*/*z* value 474.0796.
