# Supplementary material for: LC-ESI/QTOF-MS Profiling of Chicory and Lucerne Polyphenols and Their Antioxidant Activities
Source: Antioxidants (Basel). 2021 Jun 8;10(6):932. doi: 10.3390/antiox10060932 (PMC8226608; doi:10.3390/antiox10060932)
Supplement: Supplementary file 1 [file antioxidants-10-00932-s001.zip › antioxidants-1189390-supplementary.pdf]

Supplemental material

LC-ESI/QTOF-MS Profiling of Chicory and Lucerne Polyphenols and Their Antioxidant Activities

Yasir Iqbal[1], Eric N. Ponnampalam[2], Hafiz A.R. Suleria[1], Jeremy J. Cottrell[1] and Frank R. Dunshea[1,3*]

1 School of Agriculture and Food, Faculty of Veterinary and Agricultural Sciences, The University of Melbourne, Parkville, VIC 3010, Australia; yasir.iqbal@student.unimelb.edu.au (Y.I.); hafiz.suleria@unimelb.edu.au (H.A.R.S.); jcottrell@unimelb.edu.au (J.J.C.)

2 Animal Production Sciences, Agriculture Victoria Research, Department of Jobs, Precincts and Regions, Bundoora, VIC 3083, Australia; Eric.Ponnampalam@agriculture.vic.gov.au

3 Faculty of Biological Sciences, The University of Leeds, Leeds LS2 9JT, UK

* Correspondence: fdunshea@unimelb.edu.au

Abstract: Chicory and lucerne are used as specialised forages in sheep or dairy production systems in some parts of the world. Recently, these plants are gaining attention as raw materials in the search for natural antioxidants for use in animal feeds, human foods and nutraceutical formulations. The antioxidant potential of these plants is credited to polyphenols, a subgroup of phytochemicals. Therefore, phenolic characterisation is an essential step before their use as ingredients in animal feeds, human food or nutraceutical preparations. In this study, we performed qualitative and quantitative analysis of polyphenols in chicory and lucerne. Profiling of polyphenols from chicory and lucerne was performed by LC-ESI/QTOF-MS with a total of 80 phenolic compounds identified in chicory and lucerne. The quantification of polyphenols was achieved by high performance liquid chromatography, coupled with a photo diode array (HPLC-PDA). Chicoric acid was the major phenolic acid found in chicory, with the highest concentration (1692.33 ± 0.04 μg/g DW) among all the polyphenols quantified in this study. 2-hydroxybenzoic acid was the major phenolic acid found in lucerne, with the highest concentration of 1440.64 ± 0.04 μg/g DW. Total phenolic, flavonoids and total tannin contents were measured, and the antioxidant potential was determined by 2,2-Diphenyl-1-picrylhydrazyl, Ferric Reducing Antioxidant Power, 2,2-Azino-bis-3-ethylbenzothiazoline-6-sulfonic Acid, Hydroxyl (OH^-) Radical Scavenging Activity, Chelating Ability of Ferrous Ion (Fe^{+2}) and Reducing Power (RPA) assays. Both chicory (8.04 ± 0.33 mg AAE/g DW) and lucerne (11.29 ± 0.25 mg AAE/g DW) showed high values for Hydroxyl (OH^-) Radical Scavenging Activity. The current study allowed us to draw a profile of polyphenols from chicory and lucerne. They provided a molecular fingerprint useful for the application of these plant materials in human foods, animal feeds and pharmaceutical formulations.

Keywords: chicory; lucerne; polyphenols; extraction; antioxidants; LC-ESI/QTOF-MS; HPLC-PDA

Table (1S). Phenolic compounds detected and tentatively characterised in chicory by LC-ESI-QTOF/MS in both positive and negative ionisation mode

No.	Proposed compounds	Molecular Formula	RT (min)	Mode of Ionization	Molecular Weight	Theoretical (*m/z*)	Observed (*m/z*)	Mass Error (ppm)	Samples
	**Phenolic acids**								
	**Hydroxybenzoic acids**								
1	4-Hydroxybenzoic acid 4-O-glucoside	$C_{13}H_{16}O_8$	29.7	ESI + / [M+H]+	300.085	301.0918	301.093	5.31	Chicory
2	Gallic acid 4-O-glucoside	$C_{13}H_{16}O_{10}$	45.61	ESI - / [M-H]-	332.074	331.0670	331.065	-5.44	Chicory
3	Ellagic acid glucoside	$C_{20}H_{16}O_{13}$	50.4	ESI - / [M-H]-	464.059	463.0518	463.055	6.05	Chicory
	**Hydroxycinnamic acids**								
4	Cinnamic acid	$C_9H_8O_2$	12.43	ESI + / [M+H]+	148.052	149.0597	149.059	-8.05	Chicory
5	3-Caffeoylquinic acid	$C_{16}H_{18}O_9$	24.78	ESI + / [M+H]+	354.095	355.1024	355.1	-7.04	Chicory
6	Chicoric acid	$C_{22}H_{18}O_{12}$	82.6	ESI - / [M-H]-	474.08	473.0725	473.076	8.24	Chicory
	**Hydroxyphenylpropanoic acids**								
7	Dihydrocaffeic acid 3-O-glucuronide	$C_{15}H_{18}O_{10}$	28.51	ESI - / [M-H]-	358.09	357.0827	357.085	5.60	Chicory
	**Flavonoids**								
	**Anthocyanins**								
8	Cyanidin 3-O-(6''-malonyl-3''-glucosyl-glucoside)	$C_{30}H_{33}O_{19}$	28.51	ESI - / [M-H]-	697.162	696.1543	696.152	-3.88	Chicory
9	Petunidin 3-O-(6''-p-coumaroyl-glucoside)	$C_{31}H_{29}O_{14}$	49.43	ESI + / [M+H]+	625.156	626.1630	626.166	4.79	Chicory
10	Delphinidin 3-O-feruloyl-glucoside	$C_{31}H_{29}O_{15}$	51.96	ESI - / [M-H]-	641.151	640.1433	640.145	2.66	Chicory
	**Dihydrochalcones**								
11	Dihydromyricetin 3-O-rhamnoside	$C_{21}H_{22}O_{12}$	84.18	ESI - / [M-H]-	466.111	465.1038	465.104	0.65	Chicory
	**Flavanols**								
12	(-)-Epigallocatechin 3'-O-glucuronide	$C_{21}H_{22}O_{13}$	66.32	ESI - / [M-H]-	482.106	481.0987	481.101	4.16	Chicory
13	3'-O-Methyl-(-)-epicatechin 7-O-glucuronide	$C_{22}H_{24}O_{12}$	75.38	ESI - / [M-H]-	480.127	479.1195	479.121	3.97	Chicory
	**Flavanones**								
14	Hesperetin 3'-O-glucuronide	$C_{22}H_{22}O_{12}$	74.52	ESI - / [M-H]-	478.111	477.1038	477.107	6.71	Chicory
	**Flavones**								
15	Chrysoeriol 7-O-glucoside	$C_{22}H_{22}O_{11}$	46.73	ESI + / [M+H]+	462.116	463.1235	463.123	-0.86	Chicory
16	Apigenin 6-C-glucoside	$C_{21}H_{20}O_{10}$	74.52	ESI - / [M-H]-	432.1056	431.0983	431.0979	-0.93	Chicory

	**Flavonols**								
17	Kaempferol 3-O-(6''-acetyl-galactoside) 7-O-rhamnoside	$C_{29}H_{32}O_{16}$	24.68	ESI + / [M+H]+	636.169	637.1763	637.178	2.20	Chicory
18	3-Methoxysinensetin	$C_{21}H_{22}O_8$	26.7	ESI + / [M+H]+	402.132	403.1388	403.137	-5.21	Chicory
19	Isorhamnetin	$C_{16}H_{12}O_7$	29.72	ESI + / [M+H]+	316.058	317.0656	317.067	3.15	Chicory
20	Quercetin 3-O-glucosyl-xyloside	$C_{26}H_{28}O_{16}$	47.8	ESI - / [M-H]-	596.138	595.1304	595.129	-3.02	Chicory
21	Isorhamnetin 3-O-glucoside 7-O-rhamnoside	$C_{28}H_{32}O_{16}$	49.59	ESI + / [M+H]+	624.169	625.1763	625.176	-0.48	Chicory
22	Myricetin 3-O-arabinoside	$C_{20}H_{18}O_{12}$	50.27	ESI - / [M-H]-	450.08	449.0725	449.076	7.13	Chicory
23	Isorhamnetin 3-O-glucuronide	$C_{22}H_{20}O_{13}$	55.35	ESI - / [M-H]-	492.09	491.0831	491.086	5.09	Chicory
	**Isoflavonoids**								
24	6''-O-Acetylglycitin	$C_{24}H_{24}O_{11}$	7.838	ESI - / [M-H]-	488.132	487.1246	487.123	-2.87	Chicory
25	Dihydrobiochanin A	$C_{16}H_{14}O_5$	20.59	ESI - / [M-H]-	286.084	285.0768	285.076	-3.51	Chicory
	**Lignans**								
26	1-Acetoxypinoresinol	$C_{22}H_{24}O_8$	45.2	ESI - / [M-H]-	416.147	415.1398	415.139	-2.17	Chicory
	**Stilbenes**								
27	Piceatannol	$C_{14}H_{12}O_4$	7.44	ESI + / [M+H]+	244.074	245.0809	245.082	4.90	Chicory
	**Other polyphenols**								
	**Tyrosols**								
28	Oleuropein-aglycone	$C_{19}H_{22}O_8$	7.954	ESI - / [M-H]-	378.132	377.1242	377.124	-1.06	Chicory
	**Other polyphenols**								
29	Lithospermic acid	$C_{27}H_{22}O_{12}$	24.62	ESI + / [M+H]+	538.111	539.1184	539.123	7.61	Chicory

Table (2S). Phenolic compounds detected and tentatively characterised in lucerne by LC-ESI-QTOF/MS in both positive and negative ionisation mode

No.	Proposed compounds	Molecular Formula	RT (min)	Mode of Ionization	Molecular Weight	Theoretical (m/z)	Observed (m/z)	Mass Error (ppm)	Samples
	**Phenolic acids**								
	**Hydroxybenzoic acids**								
1	2,3-Dihydroxybenzoic acid	$C_7H_6O_4$	12.217	ESI + / [M+H]+	154.0266	155.0339	155.0336	-1.94	Lucerne
2	2-Hydroxybenzoic acid	$C_7H_6O_3$	13.724	ESI + / [M+H]+	138.0317	139.0390	139.0389	-0.72	Lucerne
	**Hydroxycinnamic acids**								
3	m-Coumaric acid	$C_9H_8O_3$	7.811	ESI + / [M+H]+	164.0473	165.0546	165.0548	1.21	Lucerne
4	2-S-Glutathionyl caftaric acid	$C_{23}H_{27}N_3O_{15}S$	13.954	ESI - / [M-H]-	617.1163	616.1090	616.1062	-4.54	Lucerne
5	3-Sinapoylquinic acid	$C_{18}H_{22}O_{10}$	18.196	ESI + / [M+H]+	398.1213	399.1286	399.1288	0.50	Lucerne
6	Chicoric acid	$C_{22}H_{18}O_{12}$	29.063	ESI - / [M-H]-	474.0798	473.0725	473.0696	-6.13	Lucerne
7	1,5-Dicaffeoylquinic acid	$C_{25}H_{24}O_{12}$	68.797	ESI + / [M+H]+	516.1268	517.1341	517.1319	-4.25	Lucerne
	**Hydroxyphenylpropanoic acids**								
8	Dihydrocaffeic acid 3-O-glucuronide	$C_{15}H_{18}O_{10}$	9.001	ESI - / [M-H]-	358.09	357.0827	357.0819	-2.24	Lucerne
9	Dihydroferulic acid 4-sulfate	$C_{10}H_{12}O_7S$	35.54	ESI - / [M-H]-	276.0304	275.0231	275.0218	-4.73	Lucerne
	**Flavonoids**								
	**Anthocyanins**								
10	Peonidin	$C_{16}H_{13}O_6$	24.126	ESI - / [M-H]-	301.0712	300.0639	300.0654	5.00	Lucerne
11	Delphinidin 3-O-feruloyl-glucoside	$C_{31}H_{29}O_{15}$	36.849	ESI - / [M-H]-	641.1506	640.1433	640.1413	-3.12	Lucerne
12	Pelargonidin 3-O-glucosyl-rutinoside	$C_{33}H_{41}O_{19}$	79.658	ESI - / [M-H]-	741.2242	740.2169	740.2187	2.43	Lucerne
	**Dihydrochalcones**								
13	Dihydromyricetin 3-O-rhamnoside	$C_{21}H_{22}O_{12}$	21.177	ESI + / [M+H]+	466.1111	467.1184	467.1162	-4.71	Lucerne
	**Flavanols**								
14	4'-O-Methylepigallocatechin	$C_{16}H_{16}O_7$	8.142	ESI + / [M+H]+	320.0896	321.0969	321.0986	5.29	Lucerne
15	4''-O-Methylepigallocatechin 3-O-gallate	$C_{23}H_{20}O_{11}$	19.124	ESI + / [M+H]+	472.1006	473.1079	473.1049	-6.34	Lucerne
	**Flavanones**								
16	Narirutin	$C_{27}H_{32}O_{14}$	12.463	ESI - / [M-H]-	580.1792	579.1719	579.1719	0.00	Lucerne
17	Neoeriocitrin	$C_{27}H_{32}O_{15}$	15.645	ESI + / [M+H]+	596.1741	597.1814	597.1853	6.53	Lucerne
18	Hesperetin 3',7-O-diglucuronide	$C_{28}H_{30}O_{18}$	17.136	ESI + / [M+H]+	654.1432	655.1505	655.1523	2.75	Lucerne
19	Naringenin 7-O-glucoside	$C_{21}H_{22}O_{10}$	60.018	ESI + / [M+H]+	434.1213	435.1286	435.1266	-4.60	Lucerne
	**Flavones**								
20	Luteolin 7-O-diglucuronide	$C_{27}H_{26}O_{18}$	20.714	ESI + / [M+H]+	638.1119	639.1192	639.1192	0.00	Lucerne
21	6-Hydroxyluteolin 7-O-rhamnoside	$C_{21}H_{20}O_{11}$	40.689	ESI + / [M+H]+	448.1006	449.1079	449.1073	-1.34	Lucerne

22	Apigenin 6-C-glucoside	$C_{21}H_{20}O_{10}$	41.948	ESI + / [M+H]+	432.1056	433.1129	433.1126	-0.69	Lucerne
23	Apigenin 7-O-glucuronide	$C_{21}H_{18}O_{11}$	43.107	ESI + / [M+H]+	446.0849	447.0922	447.0925	0.67	Lucerne
24	Chrysoeriol 7-O-(6''-malonyl-glucoside)	$C_{25}H_{24}O_{14}$	60.201	ESI + / [M+H]+	548.1166	549.1239	549.1236	-0.55	Lucerne
25	7,4'-Dihydroxyflavone	$C_{15}H_{10}O_4$	62.139	ESI + / [M+H]+	254.0579	255.0652	255.0647	-1.96	Lucerne
	**Flavonols**								
26	Myricetin 3-O-rutinoside	$C_{27}H_{30}O_{17}$	8.454	ESI - / [M-H]-	626.1483	625.1410	625.1404	-0.96	Lucerne
27	Spinacetin 3-O-glucosyl-(1->6)-[apiosyl(1->2)]-glucoside	$C_{34}H_{42}O_{22}$	32.757	ESI - / [M-H]-	802.2168	801.2095	801.2084	-1.37	Lucerne
28	5,4'-Dihydroxy-3,3'-dimethoxy-6:7-methylenedioxyflavone 4'-O-glucuronide	$C_{24}H_{22}O_{14}$	42.498	ESI - / [M-H]-	534.101	533.0937	533.0928	-1.69	Lucerne
29	3,7-Dimethylquercetin	$C_{17}H_{14}O_7$	44.73	ESI + / [M+H]+	330.074	331.0813	331.0812	-0.30	Lucerne
	**Isoflavonoids**								
30	3',4',5,7-Tetrahydroxyisoflavanone	$C_{15}H_{12}O_6$	19.124	ESI + / [M+H]+	288.0634	289.0707	289.0704	-1.04	Lucerne
31	3'-Hydroxygenistein	$C_{15}H_{10}O_6$	20.034	ESI - / [M-H]-	286.0477	285.0404	285.042	5.61	Lucerne
32	Puerarin	$C_{21}H_{20}O_9$	24.341	ESI + / [M+H]+	416.1107	417.1180	417.1195	3.60	Lucerne
33	Daidzein 4'-O-glucuronide	$C_{21}H_{18}O_{10}$	24.556	ESI + / [M+H]+	430.09	431.0973	431.0968	-1.16	Lucerne
34	Genistein 4',7-O-diglucuronide	$C_{27}H_{26}O_{17}$	25.186	ESI + / [M+H]+	622.117	623.1243	623.1238	-0.80	Lucerne
35	Irisolidone 7-O-glucuronide	$C_{23}H_{22}O_{12}$	26.345	ESI + / [M+H]+	490.1111	491.1184	491.1189	1.02	Lucerne
36	Tectorigenin 7-sulfate	$C_{16}H_{12}O_9S$	31.332	ESI - / [M-H]-	380.0202	379.0129	379.0147	4.75	Lucerne
37	6''-O-Malonyldaidzin	$C_{24}H_{22}O_{12}$	35.872	ESI - / [M-H]-	502.1111	501.1038	501.1035	-0.60	Lucerne
38	6''-O-Malonylgenistin	$C_{24}H_{22}O_{13}$	58.677	ESI + / [M+H]+	518.106	519.1133	519.115	3.27	Lucerne
39	2-Dehydro-O-desmethylangolensin	$C_{15}H_{12}O_4$	61.691	ESI + / [M+H]+	256.0736	257.0809	257.0801	-3.11	Lucerne
40	2',7-Dihydroxy-4',5'-dimethoxyisoflavone	$C_{17}H_{14}O_6$	64.06	ESI + / [M+H]+	314.079	315.0863	315.0855	-2.54	Lucerne
41	2'-Hydroxyformononetin	$C_{16}H_{12}O_5$	67.008	ESI + / [M+H]+	284.0685	285.0758	285.075	-2.81	Lucerne
42	3',4',7-Trihydroxyisoflavanone	$C_{15}H_{12}O_5$	81.004	ESI + / [M+H]+	272.0685	273.0758	273.0746	-4.39	Lucerne
43	6''-O-Malonylglycitin	$C_{25}H_{24}O_{13}$	82.926	ESI + / [M+H]+	532.1217	533.1290	533.1301	2.06	Lucerne
44	3'-Hydroxydaidzein	$C_{15}H_{10}O_5$	83.472	ESI + / [M+H]+	270.0528	271.0601	271.0594	-2.58	Lucerne
45	3'-Hydroxymelanettin	$C_{16}H_{12}O_6$	84.251	ESI + / [M+H]+	300.0634	301.0707	301.0699	-2.66	Lucerne
46	Dalbergin	$C_{16}H_{12}O_4$	87.066	ESI + / [M+H]+	268.0736	269.0809	269.0788	-7.80	Lucerne
	**Lignans**								
47	Sesamin	$C_{20}H_{18}O_6$	22.138	ESI + / [M+H]+	354.1103	355.1176	355.1171	-1.41	Lucerne
48	Trachelogenin	$C_{21}H_{24}O_7$	35.093	ESI - / [M-H]-	388.1522	387.1449	387.1457	2.07	Lucerne

	**Other polyphenols**								
	**Alkylphenols**								
49	4-Vinylphenol	C_8H_8O	7.894	ESI + / [M+H]+	120.0575	121.0648	121.0638	-8.26	Lucerne
	**Hydroxybenzaldehydes**								
50	4-Hydroxybenzaldehyde	$C_7H_6O_2$	31.761	ESI + / [M+H]+	122.0368	123.0441	123.044	-0.81	Lucerne
	**Tyrosols**								
51	3,4-DHPEA-AC	$C_{10}H_{12}O_4$	20.796	ESI + / [M+H]+	196.0736	197.0809	197.0809	0.00	Lucerne
52	Oleoside 11-methylester	$C_{17}H_{24}O_{11}$	64.317	ESI - / [M-H]-	404.1319	403.1246	403.1266	4.96	Lucerne
53	3,4-DHPEA-EDA	$C_{17}H_{20}O_6$	67.034	ESI - / [M-H]-	320.126	319.1187	319.1179	-2.51	Lucerne
	**Other polyphenols**								
54	Salvianolic acid D	$C_{11}H_{10}O_6$	6.085	ESI - / [M-H]-	238.0477	237.0404	237.0408	1.69	Lucerne
55	Salvianolic acid C	$C_{26}H_{20}O_{10}$	21.26	ESI - / [M-H]-	492.1056	491.0983	491.1007	4.89	Lucerne
56	Coumestrol	$C_{15}H_8O_5$	83.986	ESI + / [M+H]+	268.0372	269.0445	269.0437	-2.97	Lucerne

(a)

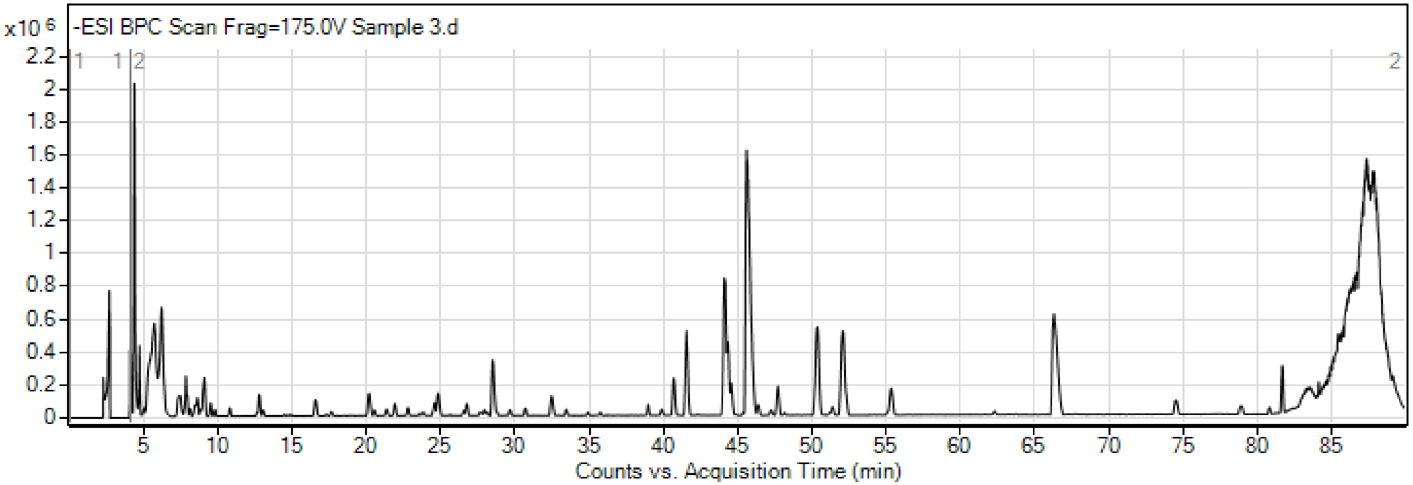
x10 6
-ESI BPC Scan Frag=175.0V Sample 3.d
2.2
2
1.8
1.6
1.4
1.2
1
0.8
0.6
0.4
0.2
0
5 10 15 20 25 30 35 40 45 50 55 60 65 70 75 80 85
Counts vs. Acquisition Time (min)

(b)

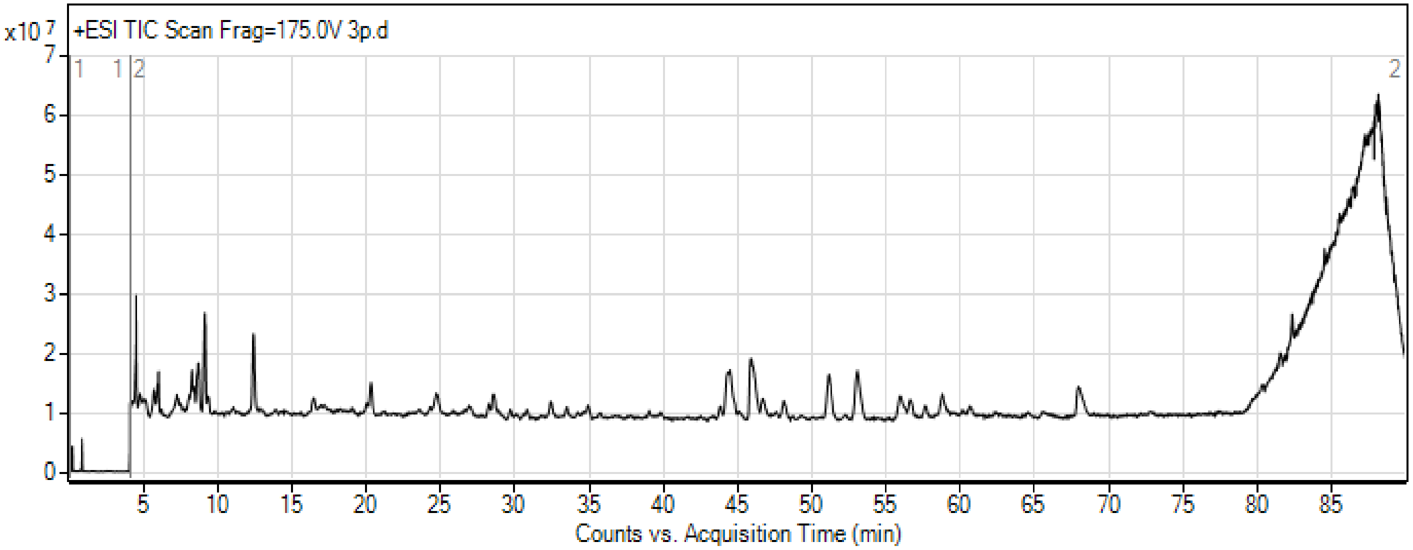
x10 7
+ESI TIC Scan Frag=175.0V 3p.d
7
6
5
4
3
2
1
0
5 10 15 20 25 30 35 40 45 50 55 60 65 70 75 80 85
Counts vs. Acquisition Time (min)

(c)

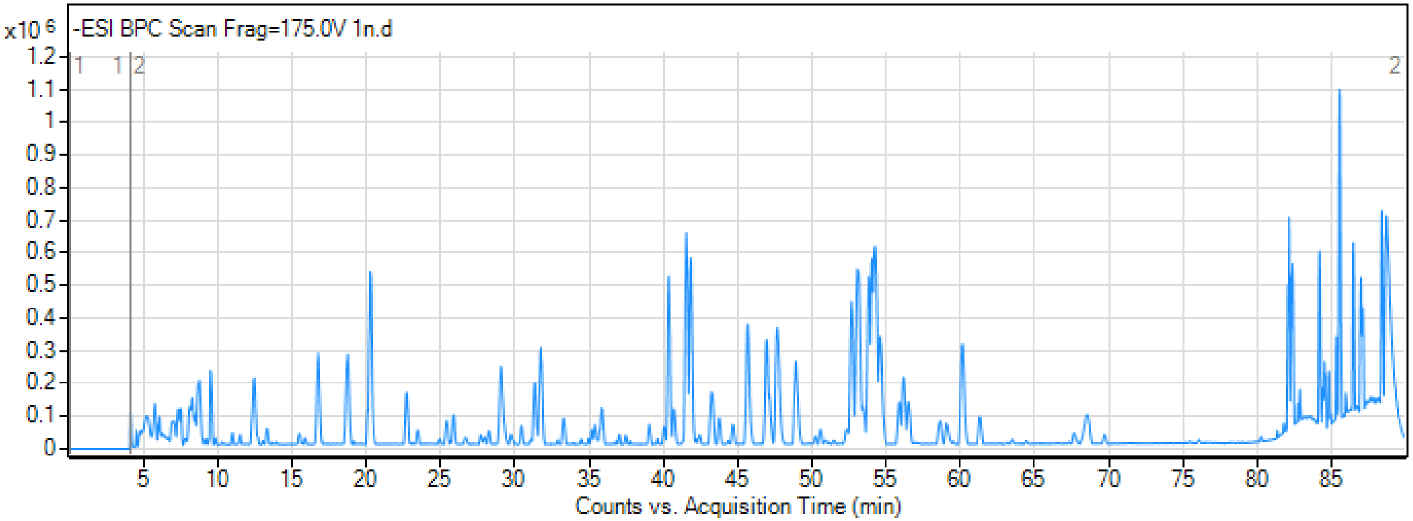

x10 6
-ESI BPC Scan Frag=175.0V 1n.d
1.2
1.1
1
0.9
0.8
0.7
0.6
0.5
0.4
0.3
0.2
0.1
0
1 1 2
2
5 10 15 20 25 30 35 40 45 50 55 60 65 70 75 80 85
Counts vs. Acquisition Time (min)

(d)

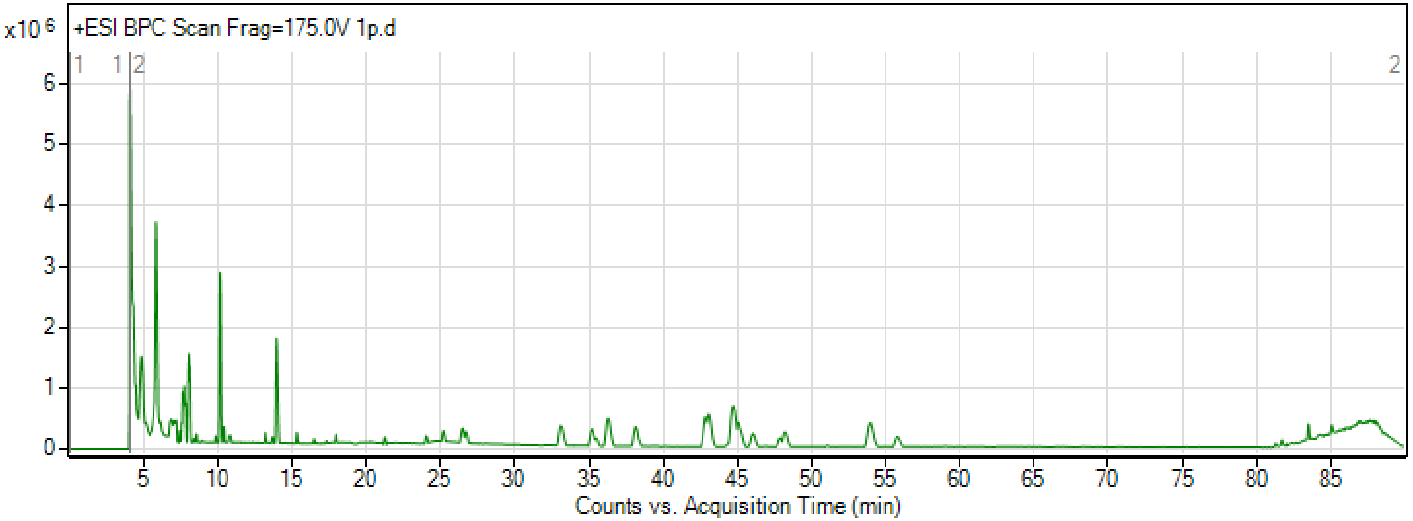

x10 6
+ESI BPC Scan Frag=175.0V 1p.d
6
5
4
3
2
1
0
1 1 2
2
5 10 15 20 25 30 35 40 45 50 55 60 65 70 75 80 85
Counts vs. Acquisition Time (min)

(e)

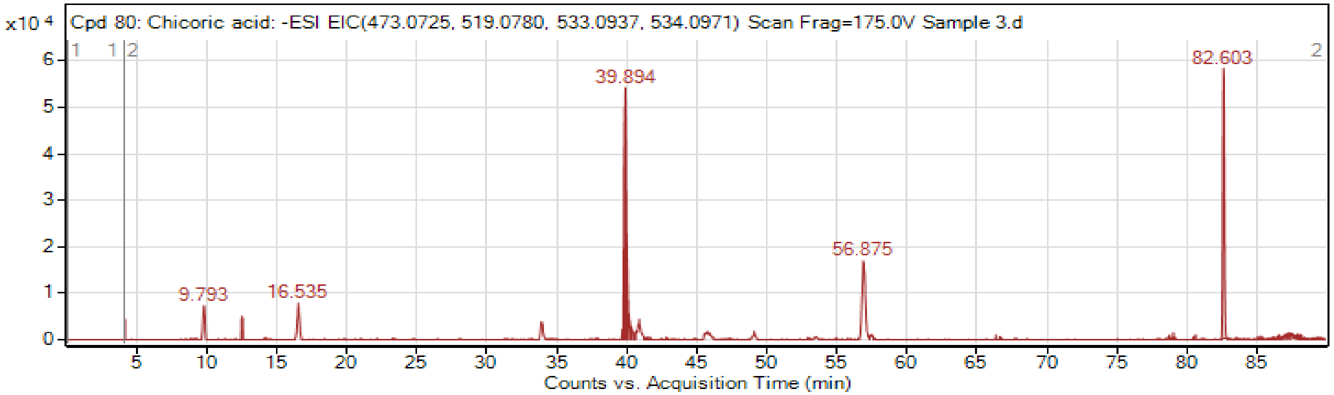

(f)

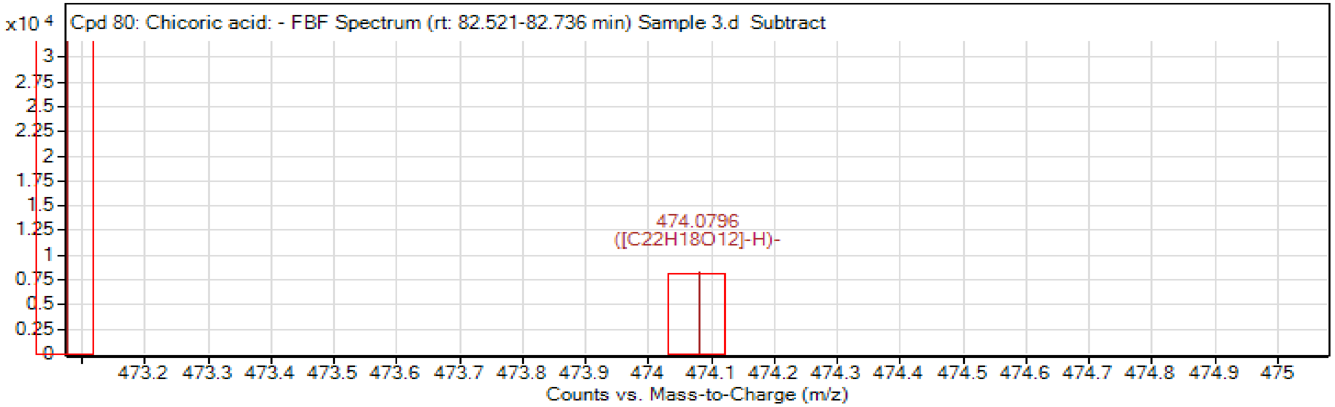

Figure (1S): LC-ESI-QTOF/MS basic peak chromatographs (BPC) for characterisation of phenolic compounds of chicory and lucerne; (a) Base Peak Chromatogram (BPC) of chicory in negative ionisation mode; (b) TIC of chicory in positive ionisation mode; (c) BPC of lucerne in negative ionisation mode; (d) BPC of lucerne in positive ionisation mode; (e) A chromatograph of chicoric acid (Compound **12** Chicory, Table 3), Retention time (RT = 82.603) in the negative mode of ionisation (ESI-/[M-H]-); (f) Mass spectra of chicoric acid showing *m*/*z* value 474.0796.
